# TB and diabetes in Eswatini: Addressing suboptimal treatment outcomes through integrated services

**DOI:** 10.1371/journal.pgph.0004607

**Published:** 2025-05-29

**Authors:** Yael Hirsch-Moverman, Dana Bezuidenhout, Sakthi Senthilvelan, Lobsang Palmo, Debrah Vambe, Ntombifuthi Ginindza, Nonhlanhla Dlamini, Andrea A. Howard, Joanne E. Mantell

**Affiliations:** 1 ICAP at Columbia University, Mailman School of Public Health, New York, United States of America; 2 Department of Epidemiology, Columbia University, Mailman School of Public Health, New York, New York, United States of America; 3 Department of Sociomedical Sciences, Columbia University, Mailman School of Public Health, New York, New York, United States of America; 4 Department of Population and Family Health, Columbia University, Mailman School of Public Health, New York, New York, United States of America; 5 Baylor College of Medicine Children’s Foundation Eswatini, Mbabane, Eswatini; 6 Department of Pediatrics, Global TB Program, Baylor College of Medicine, Houston, Texas, United States of America; 7 NCD Case Management Unit, Eswatini Ministry of Health, Mbabane, Eswatini; 8 ICAP at Columbia University, Mbabane, Eswatini; 9 Department of Psychiatry, Gender, Sexuality and Health Area, Columbia University, HIV Center for Clinical & Behavioral Studies, New York, New York, United States of America; NYU Grossman School of Medicine: New York University School of Medicine, UNITED STATES OF AMERICA

## Abstract

Tuberculosis (TB) remains a significant public health challenge in Eswatini, a country with the highest global HIV prevalence. Diabetes mellitus (DM) is increasingly common among people newly diagnosed with TB, contributing to suboptimal TB treatment outcomes, including relapse and death. The DETECT study employed a multi-method approach to assess the prevalence and DM impact on TB treatment outcomes among adults with TB in 10 Ministry of Health facilities in Eswatini, and explore barriers and facilitators to integrating TB/DM services. DM screening was conducted using laboratory-based glycated hemoglobin (A1c) testing, with DM defined as A1c ≥ 6.5% and preDM as A1c 5.7-6.4%. We conducted logistic regression to assess DM’s impact on TB treatment outcomes and 40 in-depth interviews with key stakeholders involved with TB services to explore barriers and facilitators to TB/DM service integration. Among the 373 adults diagnosed with TB and tested for DM, 13.4% had DM, 41.8% had preDM, and 44.8% had a normal A1c. The odds of poor TB treatment outcomes were 3.53 times higher (95%CI: 1.72-7.32) among those with DM compared to those without DM, after adjusting for age, HIV status, and new versus previously treated TB, highlighting the need for early DM diagnosis and treatment. Patient education and support, adequate screening resources, and community engagement were identified as facilitators of TB/DM service integration. Barriers included health system financial constraints, a perceived lack of need for exercise, insufficiently trained TB healthcare providers, inadequate supplies, societal stigma, limited patient autonomy in decision-making, and the unaffordability of healthy diets. The study found a high prevalence of DM/preDM among adults with TB in Eswatini, with DM significantly increasing the odds of poor TB treatment outcomes, irrespective of HIV status. The findings underscore the urgent need for enhanced tools and strategies to support healthcare providers in integrating TB and DM services effectively.

## Introduction

Tuberculosis (TB), HIV, and diabetes mellitus (DM) are major causes of mortality globally [1], with approximately 95% of TB, 70% of HIV, and 79% of DM cases living in low- and middle-income countries (LMICs) [1–3]. The prevalence of DM and prediabetes (preDM) is rapidly increasing in LMICs [2], particularly in regions with a high burden of TB and HIV. The global median prevalence of DM among people with TB is estimated at 16% (IQR 9%-25%) and rising obesity rates in sub-Saharan Africa are expected to further increase the prevalence of DM in this population [4]. DM is a key risk factor for both drug-sensitive and drug-resistant TB, as poorly controlled DM leads to chronic, subclinical inflammation that weakens protective immunity against *Mycobacterium tuberculosis*, the microorganism responsible for TB [5–10]. Uncontrolled DM may also negatively impact TB treatment outcomes. Poorly managed DM can cause immune dysregulation, worsening the clinical presentation of TB (e.g., increased cavitary disease and higher bacillary loads), delaying sputum culture conversion, and increasing the risk of recurrence, treatment failure, and death [9]. TB disease can also impair glycemic control in people with DM (PWDM) [11,12] and anti-TB medications can further worsen glycemic control in PWDM [13]. People living with HIV (PLHIV) are at higher risk of developing type 2 diabetes, and certain common HIV medications, like dolutegavir (DTG), may further increase this risk [14]. Despite this, few studies have prospectively examined the impact of DM on TB treatment outcomes in high TB/HIV burden settings [15].

Eswatini, a LMIC in southern Africa, has the highest HIV prevalence (24.8%) [16] globally and a high TB incidence rate (325/100,000) [1]. TB, HIV, and DM are among the leading causes of mortality in Eswatini [17]. In 2022, 65% of people with TB were also living with HIV, and faced higher mortality rates than those without HIV [1]. The 2014 World Health Organization (WHO) STEPS study in Eswatini reported that 10% of participants had impaired fasting glycemia, 44% were overweight, and 21% were obese [18]. A 2019 survey of outpatient attendees at a public health facility in Eswatini reported a prevalence of 7.3% for DM and 6.5% for preDM [19]. However, a 2016 study specifically screening PLHIV in Eswatini found a prevalence of 6% for DM and 30% for preDM [20]. Although the Eswatini National TB Control Programme (NTCP) recommends DM screening for people with TB, it is not routinely implemented [21]. The WHO also recommends integrated screening and treatment for TB/DM, especially in LMICs like Eswatini, where the prevalence of TB, HIV, and DM is high [22].

Integrated bidirectional screening and treatment services are vital in LMICs with a high prevalence of TB and non-communicable diseases (NCD) like DM [23]. Integration could enable early detection and treatment, optimize outcomes, and improve documentation, monitoring, and reporting of TB/DM cases [24]. However, there is limited knowledge about the best approach to integrated care. Some studies suggest that integrated care leads to higher screening, diagnosis, and retention rates and reduces loss to follow-up [25,26]. Yet, recent studies in Eswatini and Ethiopia have found inconsistent DM care for people with TB and suboptimal implementation of integrated services due to challenges at both the patient- and health system-levels, such as patients’ lack of financial resources [27–29], inadequate capacity of healthcare providers, frequent lab supply shortages, insufficient focus on DM care, and ineffective data management. Given Eswatini’s growing burden of DM and high TB/HIV prevalence, along with global recommendations for integrated care, it is crucial to explore these issues to understand their impact and improve treatment outcomes.

Research on the burden of DM and its impact on TB outcomes in high TB/HIV burden countries is limited, particularly when simultaneously exploring barriers and facilitators to integrated TB/DM services. This gap likely arises because these are distinct research topics often addressed through different study designs, with results integrated later. Therefore, using an observational-implementation hybrid approach (OIHA) [30], we established a prospective cohort of adults with newly diagnosed TB to estimate the burden of DM and preDM, assess the impact of DM and associated risk factors on TB treatment outcomes, and explore barriers and facilitators of TB/DM service integration in a high burden TB/HIV context.

## Methods

### Ethics statement

The protocol was approved by the Columbia University Irving Medical Center Institutional Review Board (Ref AAAT6093) and the Eswatini Health Research Review Board (Ref EHHRRB 088/2021). Both entities deemed the medical record review as eligible for waiver of individual consent. IDI participants provided written informed consent.

### Study design and setting

This multi-method, prospective cohort study was conducted at 10 public health facilities in the Manzini Region of Eswatini. As the most populous and industrialized region in the country, Manzini accounts for 45% of Eswatini’s TB cases [21]; HIV prevalence in Manzini is 26%, which is higher than the country’s prevalence [16]. The study health facilities were purposively selected in collaboration with the NTCP to ensure a representative mix of urban and rural facilities with high patient volumes. According to the Eswatini National TB guidelines, all people with TB should be screened for DM using random blood sugar or HbA1c (A1c) testing at the time of TB diagnosis, with blood pressure and body mass index (BMI) also measured. Prior to study implementation, study staff reminded healthcare providers (HCP) at participating facilities to conduct DM screening promptly after a TB diagnosis as per national guidelines.

### DM/preDM prevalence and impact on TB treatment outcomes

#### Participants.

The prospective cohort included all adults aged ≥18 years who were newly registered for TB treatment and screened for DM using A1c between June 2022 and February 2023 at participating health facilities.

#### Data collection.

Study staff supported NTCP healthcare providers in delivering standard of care by ensuring that they collected blood specimen for A1c testing at the time of TB diagnosis. To confirm that DM status accurately reflects the condition at the time of TB diagnosis, we included only lab-based A1c tests conducted within two months of diagnosis. PreDM was defined as 5.7% ≥ A1c<6.5% and DM as A1c≥6.5% [31]. Patients with a prior DM diagnosis were classified as having DM regardless of their A1c at TB diagnosis. Trained research assistants retrospectively abstracted demographic (age, sex) and clinical data (A1c, blood pressure, BMI, HIV status, site and extent of TB disease, and TB treatment outcomes) from medical records between 01/06/2022 and 30/11/2023 for research purposes. SurveyCTO was used for quantitative data collection and entry.

#### Data analysis.

The burden of DM/preDM among adults with TB was measured by the percentage of adults with TB who screened positive for DM/preDM. Poor TB treatment outcomes were defined as positive sputum smear or culture at five months or later [32], loss to follow-up, or death. We compared treatment outcomes of patients with DM and without DM (preDM or TB only) using Pearson’s Chi-square test. To examine the impact of DM on TB treatment outcomes, we conducted bivariate logistic regression. Factors associated with the outcome (p-value ≤ 0.20) were included in the multivariable model. Multivariable logistic regression with backward selection was used to estimate the odds of TB treatment failure based on DM status and other covariates, including known risk factors for TB treatment failure or death, such as HIV status, BMI, and age. The interaction effect between DM and HIV was also examined. Statistical significance was determined using a two-tailed alpha level of 0.05. We performed all analyses with STATA 18 software (StataCorp, College Station, Texas, USA).

### Barriers and facilitators of TB/DM service integration

#### Participants.

Two groups of stakeholders were enrolled in the qualitative research component: HCPs and key informants (KIs). In-depth interview (IDI) inclusion criteria included individuals aged ≥18 years, siSwati- or English-speaking, and willing to have the interview audio-recorded. HCP participants were doctors or nurses providing TB services at Ministry of Health (MOH) facilities in Manzini while KI participants were managers in the TB, AIDS, or NCD programs. We purposively sampled HCP participants from MOH facilities offering TB, HIV, and/or primary care services in Manzini to ensure representation of sex, experience, and urban/rural mix.

#### Data collection.

Two trained Research Assistants experienced in qualitative research conducted the IDIs. All interviews were conducted in private, audio-recorded, transcribed verbatim, anonymized, and translated into English as needed. Qualitative data were collected using culturally sensitive, semi-structured interview guides that explored attitudes toward DM, the potential impact of DM on TB services, management of DM in people with TB, barriers to service integration, and potential intervention components for DM prevention and control (S1 Text).

#### Data analysis.

Qualitative analysis was performed using the Rigorous and Accelerated Data Reduction (RADaR) technique, a pragmatic method that uses a series of tables to triangulate themes in qualitative data and allows for a systematic approach to analyze qualitative data [33,34]. Within the rapid qualitative analysis framework, template analysis methodology was employed for efficient data analysis [35]. Two trained Research Assistants used Microsoft Excel to summarize the interview transcripts and develop template summary tables based on the structured interview guides. Template summaries were reviewed weekly with study investigators (JEM, YH-M) to ensure a systematic approach [36]. Both deductive and inductive approaches were used to create a structured yet adaptable framework for analyzing the qualitative data.

For the qualitative data analysis, we applied the revised Consolidated Framework for Implementation Research (CFIR) [37] to categorize barriers and facilitators of integrated TB/DM management and intervention strategies. Themes were identified and summarized using a deductive approach, with relevant illustrative quotes presented separately for KIs and HCPs. Sub-themes were developed to contextualize the findings. The research team reviewed and modified summary tables to reach consensus. We mapped sub-themes onto constructs aligned within four of the five CFIR domains and seven of the 39 constructs. The CFIR domains of innovation (adaptability, complexity) and inner setting (available resources) were classified as both barriers and facilitators. The domains of innovation (adaptability) and outer setting (partnerships and connections) were identified as facilitators, while the outer setting (external pressure) and individual characteristics (innovation recipients) were characterized as barriers to integrating TB/DM services (S1 Table).

### Inclusivity in global research

Additional information regarding the ethical, cultural, and scientific considerations specific to inclusivity in global research is included in the Supporting Information (S1 Checklist).

### Results

#### Prospective cohort of people with TB

**Fig 1:**
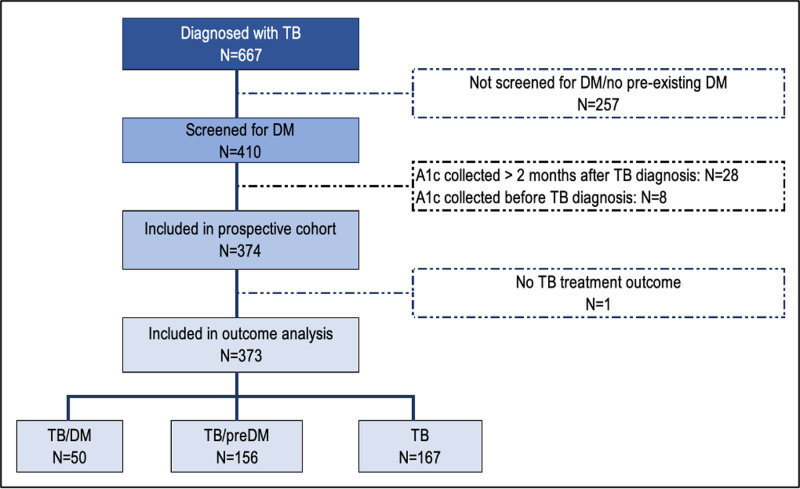
Study flow of people with TB included in the prospective cohort.

During the study period, 667 patients were diagnosed with TB across the 10 participating clinics. Among them, 15 reported pre-existing DM. Of the 667 patients, 410 were screened for DM (Fig 1), with 374 (91.2%) having an A1c test within two months of their TB diagnosis. The final analysis included 373 patients; one person was excluded due to missing TB treatment outcome data. Patients from rural clinics, with newly diagnosed TB, and favorable treatment outcomes (i.e., cured or treatment completed) were more likely to be screened for DM compared to those from urban clinics, with previously treated for TB, and with poor treatment outcomes (S2 Table).

The majority of cohort participants were from urban clinics (73.5%), male (54.0%), with a median age of 39 years (IQR: 31–47). Most patients were bacteriologically confirmed (54.4%), newly diagnosed with TB (88.4%), had pulmonary TB (87.6%), and drug-sensitive TB (96.2%). TB/HIV co-prevalence was high (76.5%), with the majority (55.2%) living with HIV for over a year, and on a DTG-based regimen (97.9%) (S2 Table).

#### DM/preDM prevalence

Among the 373 adults with TB included in the analysis, 50 (13.4%) had DM (35 newly-diagnosed DM and 15 pre-existing DM), 156 (41.8%) had preDM, and 167 (44.8) had a normal A1c (Table 1). Baseline demographic characteristics were comparable across the three groups, except for clinic location, age, TB patient type, and TB treatment outcome. Individuals with DM were more frequently from urban clinics, older, and more often diagnosed as new TB patients compared to those with preDM and no DM. Additionally, they were more likely to have a poor TB treatment outcome.

**Table 1:** Comparison of baseline characteristics of those with TB only, prediabetes and TB, and diabetes and TB.

Patient Characteristics		TB Only *(167, 44.8%)*		Pre-Diabetes/TB *(156, 41.8%)*		Diabetes/TB *(50, 13.4%)*		P-value
Clinic Location	Rural	37	*22.2%*	52	*33.3%*	10	*20.0%*	**0.040**
	Urban	130	*77.8%*	104	*66.7%*	40	*80.0%*	
Sex	Female	86	*51.5%*	64	*41.0%*	22	*44.0%*	0.195
	Male	81	*48.5%*	92	*59.0%*	28	*56.0%*	
Age (median, IQR)		37	*29-45*	39	*31-48*	47	*29-45*	**0.001**
Smoking History	No smoking history	28	*80.0%*	23	*85.2%*	6	*85.7%*	0.896^†^
	Smoking history	7	*20.0%*	4	*14.8%*	1	*14.3%*	
BMI	Normal (≤24.9 kg/m^2^)	124	*78.5%*	123	*80.4%*	31	*67.4%*	0.171
	Overweight/obese (>25.0 kg/m^2^)	34	*21.5%*	30	*19.6%*	15	*32.6%*	
Hypertension^	Normal blood pressure	74	*84.1%*	65	*77.4%*	24	*75.0%*	0.412^†^
	Hypertension	14	*15.9%*	19	*22.6%*	8	*25.0%*	
HIV Status	Negative	35	*21.0%*	41	*26.3%*	12	*24.0%*	0.529
	Positive	132	*79.0%*	115	*73.7%*	38	*76.0%*	
HIV Diagnosis	New HIV diagnosis	47	*40.2%*	50	*50.0%*	15	*45.5%*	0.348
	Established HIV	70	*59.8%*	50	*50.0%*	18	*54.5%*	
ART Regimen	DTG-based regimen	83	*95.4%*	80	*100.0%*	26	*100.0%*	0.154^†^
	Non-DTG-based regimen	4	*4.6%*	0	*0.0%*	0	*0.0%*	
CD4, cells/µl (median, IQR)		200	*73-410*	93	*44-324*	93.5	*62-265*	0.146
TB Patient Type	New patient^§^	136	*82.9%*	144	*92.3%*	48	*96.0%*	**0.008** ^†^
	Previously treated^‡^	28	*17.1%*	12	*7.7%*	2	*4.0%*	
Type of TB Diagnosis	Bacteriologically confirmed	90	*57.7%*	77	*51.7%*	26	*53.1%*	0.560
	Clinical Diagnosis	66	*42.3%*	72	*48.3%*	23	*46.9%*	
Site of TB	Extrapulmonary	24	*14.6%*	12	*7.7%*	9	*18.4%*	0.051
	Pulmonary	140	*85.4%*	144	*92.3%*	40	*81.6%*	
TB Drug Sensitivity	Drug-resistant	6	*3.6%*	4	*2.6%*	3	*6.0%*	0.496^†^
	Drug-sensitive	160	*96.4%*	152	*97.4%*	47	*94.0%*	
TB Treatment Outcomes	Poor outcome*	27	*16.2%*	12	*7.7%*	17	*34.0%*	**0.000**
	Cure/treatment completion	140	*83.8%*	144	*92.3%*	33	*66.0%*	

^Hypertension was defined as systolic blood pressure ≥ 130 mmHg or diastolic blood pressure ≥ 80 mmHg.

§A person with tuberculosis who has never received treatment or has only previously ever taken anti-tuberculosis drugs for less than 1 month.

‡Previously treated includes patients with relapse, treatment failure, loss to follow up, or transfer from another facility.

*Poor outcome included those who died, were lost to follow-up, had treatment failure, and were not evaluated.

Observations not available for the following: Smoking history: N = 304, BMI: N = 16, Hypertension: N = 169, HIV Diagnosis: N = 35; CD4: N = 166, Patient Type: N = 3, Type of TB Diagnosis: N = 19; Site of TB: N = 4, TB Drug Sensitivity: N = 1.

†Fisher’s exact test.

#### DM impact on TB treatment outcomes

In the prospective cohort, 85.0% of patients were cured or completed TB treatment (S3 Table). However, patients with DM — whether pre-existing or newly diagnosed — were significantly more likely to have poor TB treatment outcomes compared to those without DM (OR: 3.75; 95% CI: 1.91-7.36). Additionally, age, HIV status, and TB patient type were independently associated with poor TB treatment outcomes (Table 2). After adjusting for these factors, the odds of a poor TB treatment outcome among those with DM were 3.53 times higher (95% CI: 1.72-7.32) compared to those without DM (Table 2).

**Table 2:** Bivariate and multivariable logistic regression analyses for poor TB treatment outcome (n = 373).

	Patient Characteristics		Bivariate			Multivariable w/ backward selection (N = 370)	
		OR	95% CI	P-value	OR	95% CI	P-value
Diabetes	DM*	3.75	1.91-7.36	<0.001	3.53	1.72-7.23	<0.001
	No DM		REF			REF	
BMI	Overweight/obese (>25.0)	1.37	0.68-2.73	0.38			
	Normal(<24.9 kg/m^2^)		REF				
Clinic Location	Urban	1.10	0.57-2.11	0.78			
	Rural		REF				
Sex	Female	0.79	0.44-1.40	0.41			
	Male		REF				
Age (median, IQR)		1.04	1.02-1.06	<0.001	1.04	1.01-1.06	<0.001
HIV Status	Positive	2.03	0.92-4.46	0.08	2.36	0.99-5.59	0.05
	Negative		REF			REF	
TB Patient Type	Previously treated^‡^	2.29	1.07-4.89	0.03	2.61	1.17-5.82	0.02
	New patient^§^		REF				
Type of Diagnosis	Bacteriologically confirmed	0.47	0.25-0.85	0.01			
	Clinical diagnosis		REF				
Site of TB	Pulmonary	0.47	0.22-1.00	0.05			
	Extrapulmonary		REF				
TB Drug Sensitivity	Drug-sensitive	2.17	0.28-17.04	0.46			
	Drug-resistant		REF				

*DM includes those with newly diagnosed DM or pre-existing DM.

‡Other patients were those who were previously treated due to relapse, treatment failure, loss to follow-up, or transfer from another facility.

§A person with tuberculosis who has never received treatment or has only previously ever taken anti-tuberculosis drugs for less than 1 month.

#### Barriers and facilitators of TB/DM service integration

**Fig 2:**
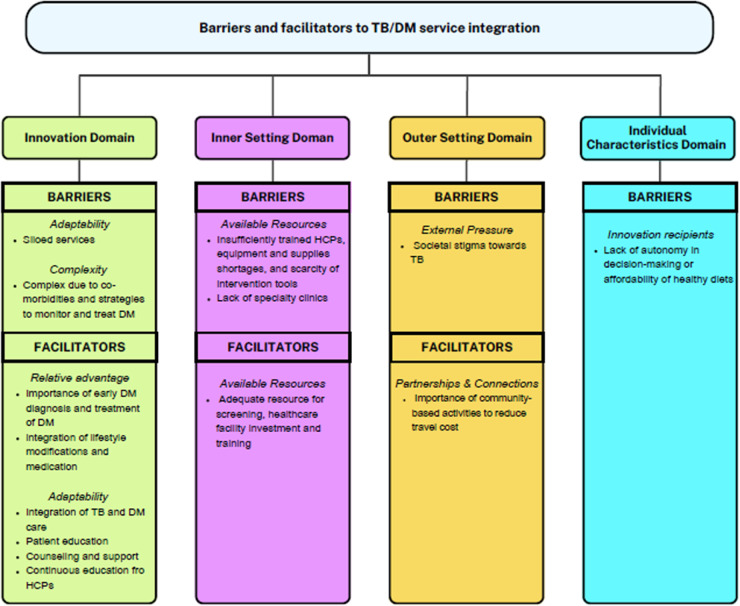
Perceived barriers and facilitators to TB/DM service integration with revised CFIR.

A total of 30 interviews with HCPs and 10 with KIs were conducted. The average interview duration was about 35 minutes (SD ± 12.73 for HCPs and ±5.68 for KIs). On average, HCPs had 5.6 years (±4.9) of experience working in TB facilities, while KIs had an average of 5.2 years (±4.0) of experience. Fig 2 provides a summary of barriers and facilitators to TB/DM service integration.

#### Barriers

Key barriers included siloed services, complexity of DM management, insufficient training of HCPs, inadequate equipment and supplies such as glucose strips, societal stigma associated with TB, lack of specialty clinics, and limited patient autonomy in decision-making or affordability of healthy diets (Table 3).

**Table 3:** Perceived barriers to TB/DM service integration categorized by the revised CFIR.

CFIR Constructs	Findings	Illustrative Quotes	
		*Key Informants*	*Healthcare Providers*
**Innovation**			
**Adaptability**	Siloed services	“… there is no integration yet for what I have seen so you find that TB patients are just treated for TB… maybe they are tested for HIV… HIV patients are being tested and treated for TB and TB patients are tested and treated for HIV.” (00-KI-01)	“…we just need to integrate the care for diabetes and TB because it seems like it is something which was being neglected.” (06-HP-001)
**Complexity**	Complex due to co-morbidities and strategies to monitor and treat DM	“It [DM treatment] is complex …because we need to constantly monitor the client there’s lot of control that we need to do or before we have to monitor or when we are trying to control the blood because it also includes what the food the client is also taking, the lifestyle, so it is complex and need to monitor constantly...” (00-KI-03).	“I think it becomes complex when we now talking about injection. But taking the tablets is just manageable.” (09-HP-003) “What makes it complex is the comorbidity... Because most of patients, they have DM and hypertension, DM and urine failure, DM and HIV, DM and TB.” (05-HP-003)
**Inner Setting**			
**Available resources**	Insufficiently trained HCPs, equipment/ supply shortages, & scarcity of intervention tools.	“… lack of the equipment like glucometer machines…at times the glucometer would be there but there will be no tests strips so those challenges with the equipment.” (00-KI-04) “…we do not have enough TB screening tools, so it becomes a challenge…” (00-KI-04)	“… the human resource, one, is of great importance because we miss due to insufficient human resource. Two, the resources that we need: the screening tools, trainings that should be done to capacitate those people.” (02-HP-001)
	Lack of specialty clinics	“The feeling is that there are no healthcare providers that specialize in diabetes…the nurses that work in the TB department also work in other departments. The healthcare workers are sometimes overwhelmed by the number of patients they have to attend to…” (00-KI-09)	N/A
**Outer Setting**			
**External pressure**	Societal stigma towards TB	“… we [are] trying to give the service to the community and they say ‘no no no, people are stigmatizing...’” (00-KI-07) “… usually people are associating TB screening mainly with stigma to other communicable disease such as HIV so there’s always that stigma …” (00-KI-03)	“… you know TB has got stigma here in the country. People they think ‘Hey, you are taking TB treatment and now they are screening you.’ They won’t be comfortable. The people who have diabetes are not going to be comfortable if you start digging in and then you are asking them all those questions pertaining TB with still the stigma, …” (01-HP-003).
**Individual Characteristics**			
**Innovation recipients**	Lack of autonomy in decision-making	N/A	“...they don’t have a choice sometimes because especially when it comes to the diet part, some of them, they are in the family where they just eat the pot that is preparing in the pan, so they cannot make their own decision.” (05-HP-003)
	Unaffordability of healthy diets	N/A	“And some of them, maybe they can’t afford. They are working and they just eat what is presented at the workplace. They cannot actually make a decision.” (05-HP-003)

##### Innovation domain.

*Adaptability.* The innovation domain refers to TB/DM service integration, which may include screening for DM at TB clinics or screening for TB at DM clinics with linkage between programs. Alternatively, service integration could consist of a one-stop model in TB clinics, where patients receive care for both TB and DM, similar to the integration of TB/HIV services. Both HCPs and KIs reported that the system was quite siloed and noted a need for integrating services, illustrating with the example of TB/HIV services.

*Complexity* of DM treatment was described as multifactorial. Once DM is diagnosed, regular monitoring of blood sugar levels is essential, but fluctuations can occur due to medications and diet, leading to random spikes even when patients adhere to their treatment regimens. Another complexity is the variety of treatment options, where some oral medications may be more effective or manageable than others, such as insulin injections. Furthermore, selecting an appropriate treatment that does not conflict with the TB treatment is challenging, especially as many times patients present with multiple co-morbidities.

##### Inner setting domain.

*Available resources.* Human resources, equipment, supplies, and intervention tools are essential for the effective implementation of interventions. However, both HCPs and KIs reported challenges in these areas, including insufficiently trained HCPs, shortages of equipment and supplies, and a scarcity of intervention tools, such as screening algorithms and patient education materials. In addition, a lack of HCPs that specialize in DM was noted.

##### Outer setting domain.

*External pressure.* Both HCPs and KIs highlighted that individuals with TB/DM often face discrimination due to societal stigma surrounding TB. This stigma leads to reluctance in seeking screening and treatment.

##### Individual characteristics domain.

*Innovation recipients.* HCPs identified challenges related to healthy diet choices for individuals with DM, noting that a lack of autonomy in decision-making and the affordability of healthy foods are significant barriers.

#### Facilitators

Key facilitators included the importance of early DM diagnosis and treatment, integrating lifestyle modifications with medication; and the perceived simplicity of DM treatment when lifestyle choices are understood. Additional facilitators were patient education, support groups, ongoing education for HCPs, adequate resources for screening and training, and the crucial role of community engagement (Table 4).

**Table 4:** Perceived facilitators to TB/DM service integration categorized by the revised CFIR.

CFIR Constructs	Findings	*Illustrative Quotes*	
		*Key Informants*	*Healthcare Providers*
**Innovation**			
**Relative advantage**	Importance of early diagnosis and treatment of DM	“…at times, you wouldn’t know the patient has diabetes, you would just treat the TB only and you find that the diabetes ends up killing the patient so I think it would help in preventing mortality and improving TB treatment outcomes.” (00-KI-04)	“…we can diagnose diabetes at an early stage and begin to manage it because when it is diagnosed at a later stage, it might lead to a complication of TB.” (02-HP-001)
	Integration of lifestyle modifications and medication	“…tell them [patients] the importance of changing their lifestyle because even if you can put them on treatment and their lifestyle doesn’t change…it won’t help much because medication and lifestyle should go together…” (00-KI-08)	“…we should include lifestyle, dietary and the medical. So, we shouldn’t just focus on the medication only…if we can combine those, we can reach normal glucose levels.” (03-HP-004).
**Adaptability**	Integration of TB/DM services	“…people know diabetic clinic and TB clinics as separate entities…so running those systems in silos would go against integration so I think just an effort towards integrating them would improve outcomes…” (00-KI-10)	“…we just need to integrate the care for diabetes and TB because it seems like it is something which was being neglected.” (06-HP-001) “… would help in diagnosing pre-diabetes before it actually became full blown diabetes. I think it would help in missing diagnoses… early detection and information would improve outcomes and then probably also improve adherence to medication as well.” (06-HP-003) “We would need an in-service training on how to take care of TB plus Diabetes because right now we do have information about TB/HIV…right now we just need an in-service training … a workshop on how to take care of those clients “ (03-HP-001)
	Patient education	“...you also need to educate the audience in lay man’s terms so that they will be able to understand the section and …opportunities and ideas on how best to manage their condition. …” (00-KI-01)	“…overall pamphlet containing information about diabetes. The do and the don’t is very vital …it helps these people to understand what they need to do.” (06-HP-002)
	Counselling and support	“… counselling works … when we have issue of pill burden… and also some stigma, we really need to invest in counselling and outcomes for these patients of TB…” (00-KI-07) “…allowing them to form support groups so that the peer education is very crucial and very important…when a patient is sick and looks at another patient …they can share ideas together…how they manage their disease together.” (00-KI-08)	“…if you have a support group of people with similar conditions who could sit and discuss... so they don’t feel alone.” (06-HP-003)
	Continuous education for HCPs	“[CME]‘s a brilliant idea, in fact in our organization we are writing a concept to try and recommend this to the Medical Council in Swaziland, so that we have a standardized CME for doctors and other care providers.” (00-KI-01)	“I’d like to receive information about the relationship between diabetes and TB. …I want to know more about pre-diabetes and management of diabetes, especially the drugs…” (04-HP-003)
**Inner Setting**			
**Available resources**	Adequate resources for DM screening to provide quality services	“... let’s invest in the resources… I mentioned the issue of self-screening for some clients that are able and also improve access to screening services themselves… it comes to the issue of resources...” (00-KI-01)	“… the resources that we need: the screening tools, trainings that should be done to capacitate those people.” (02-HP-001)
**Outer Setting**			
**Partnerships & connections**	Importance of community-based activities to reduce transport cost for clinic visits	“... we need to look into community-based activities so be it support groups at community levels... be it some level of community-based NCD [non-communicable disease] support from the facility to meet the patients halfway so that reduces also the costs that are associated with NCDs... “ (00-KI-10)	N/A

##### Innovation domain.

*Relative advantage.* Both KIs and HCPs frequently emphasized the importance of early diagnosis and treatment of DM in the success of integrated TB/DM services, including preventing complications and managing DM-related health issues. KIs further noted that early detection and treatment of DM can significantly reduce mortality in people living with TB. Both groups also underscored the importance of combining lifestyle modifications with medication for effective DM management, as medication alone may not suffice and should be combined with healthy diets to stabilize blood sugar levels and maintain desired weight. Some HCPs even emphasized that lifestyle changes could be more important than medication alone in managing DM.

*Adaptability* was seen as a positive factor in managing TB/DM treatment, allowing for greater flexibility in healthcare services. Both HCPs and KIs emphasized the importance of integrated TB/DM services, where HCPs trained in both conditions can provide comprehensive care, leading to better outcomes. They also highlighted the need for patient education on DM management, particularly using simple language to ensure comprehension. Providing educational materials, such as pamphlets in both siSwati and English, was viewed as an effective strategy. Additionally, KIs and HCPs believed that sharing health challenges could foster a sense of support among patients. HCPs called for continuous education on the relationship between TB and DM, along with training and mentorship on how to care for people with both conditions to enhance service quality.

##### Inner setting domain.

*Available resources.* Both KIs and HCPs pointed out the challenges of delivering quality services without adequate resources for DM screening, underscoring the need for investment in healthcare facilities and training in this area.

##### Outer setting domain.

*Partnerships and connections*. The community plays a vital role in organizing community-based activities and TB/DM programs, which helps bring healthcare services closer to the community and reduces patients’ transport costs to clinics.

### Discussion

The rapidly growing epidemic of DM in LMICs poses a significant threat to TB control efforts. However, few studies have prospectively examined the impact of DM on TB treatment outcomes in high TB/HIV burden settings while also exploring barriers and facilitators to integrated service delivery. Early diagnosis of DM during TB treatment is crucial, enabling the integration of DM services into TB/HIV care. Understanding the prevalence and incidence of DM, its impact on TB outcomes, and associated risk factors, alongside exploring diverse stakeholder preferences, is essential for tailoring interventions and improving TB treatment success.

We prospectively followed 373 adults with TB, with and without DM, in a high TB/HIV prevalence setting. Our findings revealed that individuals with DM were more likely to experience poor TB treatment outcomes compared to those without DM, even after adjusting for age, HIV status, and TB patient type. Using the CFIR, we identified key barriers and facilitators to TB/DM service integration. Significant barriers included health system financial constraints, perceived lack of need for exercise, insufficiently trained HCPs, inadequate supplies, societal TB stigma, limited patient autonomy in decision-making, and the affordability of healthy diets. Facilitators included early diagnosis and treatment of DM, patient education and support, adequate screening resources, and community engagement.

Contrary to several existing studies and systematic reviews, our study found substantially higher odds of poor TB treatment outcomes among people with TB/DM compared to those without DM [9,38]. A recent meta-analysis reported that people with comorbid TB/DM had 1.65 times (95%CI 1.39-1.96) odds of poor TB treatment outcomes (death or treatment failure) compared to those with only TB [9]. In contrast, our study found an adjusted odds ratio of 3.52 (95%CI 1.72-7.32), suggesting much greater odds. However, our study likely underestimated the odds ratio due to selection bias, as those with poor TB treatment outcomes or previously treated for TB were less likely to be included in the prospective cohort (Table 1). These higher odds may be attributed to the study context, as only two studies in the meta-analysis were conducted in LMICs with high TB/HIV burden and reported similar findings to our context [39,40]. Although we found a larger effect of DM on TB treatment outcomes compared to previous research, our findings regarding risk factors for poor TB treatment outcomes were consistent. Specifically, older age [41], living with HIV, and a history of previously treated TB were associated with poor outcomes.

The qualitative findings provided valuable insights into HCPs’ and KIs’ perspectives regarding the benefits of TB/DM service integration in Eswatini. Participants emphasized that early DM diagnosis and treatment were key to preventing complications and managing DM-associated health issues. Similar findings have been reported in studies from Malawi and India, which highlighted the benefits of integrated TB/DM services for better treatment outcomes [25,26]. Given DM’s negative influence on TB treatment outcomes [9,10], early detection through an integrated approach was found to be instrumental in mitigating potential risks and complications [42].

Participants also stressed the importance of a holistic approach to DM management, integrating lifestyle modifications and medication. They recognized that lifestyle modifications, such as consistent physical exercise and effective weight management, and following HCPs’ advice on maintaining a healthy diet, play a crucial role in reducing blood sugar levels. These findings align with a recent study from Eswatini and a systematic review conducted in Africa, both emphasizing the importance of lifestyle interventions in preventing and managing DM. However, barriers at both the system and personal levels were identified, including limited access to services, the influence of western culture, poverty, and low levels of education [28,43].

Patient education emerged as another key theme, with participants highlighting the need for clear, accessible communication to help patients understand and manage their condition. This was consistent with a study in Ethiopia, which identified poor knowledge and financial constraints as barriers to dietary adherence [44]. Our study suggests that providing educational materials in siSwati and English, along with straightforward language, could help overcome these barriers.

Community involvement was seen as essential, with participants advocating for community-based support groups for patients to share their health challenges and foster a sense of support. They also advocated for initiatives that bring health services closer to patients, reducing the burden of travel to multiple or distant clinics. These findings echo a study in Uganda, where the establishment of an HIV/NCD integrated clinic reduced transportation barriers and improved access to healthcare for HIV and NCDs, making services more accessible and affordable for patients [45].

Participants discussed the multifactorial nature of DM treatment as individuals with DM experience an elevated susceptibility to lung infections, and the likelihood of more severe TB disease when coexisting with DM [46]. Insufficiently trained HCPs, shortages of equipment, a lack of essential supplies and a scarcity of intervention tools such as screening algorithms, patient education materials, as well as regular HCP training and mentorship, were described as potential barriers to managing TB/DM service integration. Notably, patients from rural clinics were more likely to be screened for DM, suggesting that smaller clinics may be more inclined to incorporate DM screening compared to large, urban clinics. Our study findings were consistent with prior studies that reported the shortage of qualified HCPs and adequate equipment and supplies were barriers to delivering services for NCDs [28,47,48]. The importance of human resources and lack of essential equipment and supplies, e.g., glucometers and test strips, was emphasized, particularly in the context of screening patients in similar settings [28,47]. In South Africa, malfunctioning blood pressure measurement devices were perceived to be a barrier to delivering effective, integrated, high-quality chronic disease management [49]. Therefore, a study in sub-Saharan Africa recommended that governments in Africa strengthen their healthcare systems to address the rising prevalence of NCDs such as DM [50].

Finally, participants highlighted the societal stigma surrounding TB, which discourages individuals from seeking screening and treatment. This aligns with other studies that have identified stigma as a barrier to integrated NCD care [51,52]. However, a Ugandan study reported that HIV-related stigma was greatly reduced after the integration of HIV/NCD care, suggesting that integrated services may help alleviate some of these challenges due to participants’ comfort in sharing their experiences and increased understanding of the diseases [45].

#### Study strengths and limitations

A significant strength of this study was the use of the OIHA framework to prioritize the expedited translation of findings into public health impact [30], particularly for underserved populations such as adults with TB in Eswatini. OIHA can integrate implementation science methods into observational studies to anticipate or infer the effects of interventions and implementation strategies. This approach may reduce the research pipeline’s time and enhance the study’s practical relevance. For instance, this framework allowed us to simultaneously assess the impact of DM on TB treatment outcomes and identify barriers and facilitators to service integration, a process that traditional research methods would typically address in separate research studies.

The study’s focus on a high TB/HIV burden setting, where research is limited, and the use of A1c testing for DM diagnosis, also strengthened its findings. A1c testing, endorsed by WHO in 2011, is relatively stable and does not require fasting, making it feasible to diagnose DM once TB is identified. Additionally, the study provided an in-depth exploration of the challenges and opportunities associated with integrating TB/DM services, drawing on the perspectives of HCPs and KIs with experience in TB/DM service provision. The systematic application of an implementation science theoretical framework (i.e., CFIR) enhanced the clarity and scientific rigor of the study’s findings by offering a structured approach to evaluating these perspectives while considering the context and enabling a theory-informed analysis that strengthens the interpretation and applicability of the results.

Despite these strengths, the study also had some limitations. First, it was conducted in one region (Manzini) of Eswatini, which may limit its generalizability. However, Manzini’s large population, high TB burden, and mix of urban and rural clinics make it a relevant setting. Second, we were unable to assess transient DM in this study, which could inflate the burden of DM in this population. However, the significantly worse TB treatment outcomes among people with TB/DM suggest that transient DM was not a major factor. Third, while Eswatini’s guidelines recommend random blood sugar testing for DM screening, we used A1c testing to ensure diagnostic accuracy and align with WHO guidelines. Although A1c testing may be less accurate in patients with anemia or other conditions affecting red blood cell turnover [53], our previous study found minimal anemia (M Rabkin, personal communication) among PLHIV screened for DM using A1c in Eswatini. This new testing procedure may have contributed to the exclusion of one-third of the TB patients who were not tested for DM or were not tested within two months of TB diagnosis. Fourth, while the study used a prospective design, demographic and clinical data were retrospectively abstracted from medical records. As a result, some data on key risk factors were missing, potentially influencing the estimation of the effect of DM on TB treatment outcomes. For example, smoking status was not consistently recorded in medical records, preventing its inclusion in our analysis. Smokers are more likely to develop DM than non-smokers [54], and smoking has been associated with an increased likelihood of poor TB treatment outcomes [55]. Study staff made efforts to capture complete information by cross-referencing data from medical records and TB registries, however this limitation may have overestimated the impact of DM on TB treatment outcomes. Lastly, although we applied CFIR to identify barriers and facilitators to TB/DM service integration, the study was not designed with CFIR in mind, which could introduce some limitations.

### Conclusion

The DETECT study underscores the need for comprehensive strategies to effectively address barriers and facilitators to TB/DM service integration. These strategies are essential for minimizing the risks associated with poor outcomes of both diseases and streamlining service delivery by consolidating them within a single clinic. Proposed implementation strategies include the establishment of standardized operating procedures for TB/DM services, community health education activities, training of community health workers and HCPs, and task shifting to facilitate the provision of TB/DM services. Additionally, interventions to reduce stigma surrounding TB/DM can be achieved through community engagement, continuing education, and the creation of supportive environments for individuals living with TB/DM comorbidities. Enhancing HCP training, improving access to essential equipment, and strengthening healthcare systems are essential for the evidence-based integration of TB/DM services.

### Supporting information

S1 Table Operationalized definitions of CFIR domains and constructs. (DOCX)

S2 Table Comparison of baseline characteristics of prospective cohort vs. those not tested for DM. (DOCX)

S3 Table Comparison of baseline characteristics of those with cure/treatment completion vs. poor outcomes. (DOCX)

S1 Text Qualitative interview guides (healthcare providers and key informants). (PDF)

S2 Text Quantitative codebook. (PDF)

S1 Checklist Inclusivity in global research. (DOCX)

S1 Data Quantitative dataset. (CSV)

S2 Data Qualitative dataset. (XLSX)
