# Supplementary material for: TB and diabetes in Eswatini: Addressing suboptimal treatment outcomes through integrated services
Source: PLOS Glob Public Health. 2025 May 29;5(5):e0004607. doi: 10.1371/journal.pgph.0004607 (PMC12121823; doi:10.1371/journal.pgph.0004607)
Supplement: S1 Table — (DOCX) [file pgph.0004607.s001.docx]

**S1 Table.** Operationalized definitions of CFIR domains and constructs

| **CFIR Domains & Constructs** | **Definitions** |
| --- | --- |
| **Innovation** | Aspects of an innovation that may impact success of implementation, including strategies of managing TB/DM, nature of the disease and its treatment. |
| Relative advantage | Aspects of an innovation that may pose facilitators or barriers to implement the TB/DM service integration intervention. |
| Complexity | Aspects of an innovation that explain whether diseases and their treatments are complex or not. |
| Adaptability | Aspects of an innovation that may modify and refine the TB/DM service integration through patient and provider education. |
| **Inner Setting** | Characteristics of healthcare facilities, including availability of materials, equipment, and human resources to provide better care for people with TB/DM. |
| Available resources | Availability of resources, including adequate staff and equipment to facilitate delivering an intervention for TB/DM service integration. |
| **Outer Setting** | External factors influencing TB/DM services, including involvement with community and societal perspectives on both diseases. |
| Partnerships & connections | Involvement with communities to reduce travel cost to TB/DM clinics and reduce stigma around both diseases. |
| External pressure | External pressure such as societal stigma around TB/DM that prevents successfully delivering the intervention. |
| **Individuals Characteristics** | Characteristics and roles of individuals, including beliefs and autonomy that influence the provision of integrated services for TB/DM. |
| Innovation Recipients | Individuals living with TB/DM who are receiving the innovation. |
