# Supplementary material for: TB and diabetes in Eswatini: Addressing suboptimal treatment outcomes through integrated services
Source: PLOS Glob Public Health. 2025 May 29;5(5):e0004607. doi: 10.1371/journal.pgph.0004607 (PMC12121823; doi:10.1371/journal.pgph.0004607)
Supplement: S2 Table — (DOCX) [file pgph.0004607.s002.docx]

**S2 Table**: Comparison of baseline characteristics of prospective cohort (n=374) vs. those not tested for DM (n=293)

| **Patient Characteristics** | | **Not tested for DM  (293, 43.9*%*)** | | **Tested for DM/Existing DM  (374, 56.1*%*)** | | **P-value** | |
| --- | --- | --- | --- | --- | --- | --- | --- |
| Clinic Location | Rural | 57 | *19.5%* | 99 | *26.5%* | **0.034** |  |
|  | Urban | 236 | *80.5%* | 275 | *73.5%* |  |  |
| Sex | Female | 123 | *42.0%* | 172 | *46.0%* | 0.301 |  |
|  | Male | 170 | *58.0%* | 202 | *54.0%* |  |  |
| Age (median, IQR) | | 38 | *31-46* | 39 | *31-47* | 0.338 |  |
| Smoking History | No smoking history | 32 | *88.9%* | 58 | *82.9%* | 0.624^†^ |  |
|  | Smoking history | 4 | *11.1%* | 12 | *17.1%* |  |  |
| BMI | Normal weight (≤24.9 kg) | 213 | *80.4%* | 279 | *77.9%* | 0.459 |  |
|  | Overweight/obese (>25.0 kg) | 52 | *19.6%* | 79 | *22.1%* |  |  |
| Hypertension^ | Normal blood pressure | 105 | *71.9%* | 164 | *80.0%* | 0.078 |  |
|  | Hypertension | 41 | *28.1%* | 41 | *20.0%* |  |  |
| HIV Status | Negative | 68 | *23.2%* | 88 | *23.5%* | 0.923 |  |
|  | Positive | 225 | *76.8%* | 286 | *76.5%* |  |  |
| HIV Diagnosis | New HIV | 82 | *42.5%* | 112 | *44.8%* | 0.627 |  |
|  | Established HIV | 111 | *57.5%* | 138 | *55.2%* |  |  |
| ART Regimen | DTG-based regimen | 141 | *96.6%* | 190 | *97.9%* | 0.506^†^ |  |
|  | Non-DTG-based regimen | 5 | *3.4%* | 4 | *2.1%* |  |  |
| CD4 (median, IQR) | | 128 | *40-341* | 146 | *54-368* | 0.450 |  |
| TB Patient Type | New patient^§^ | 235 | *82.2%* | 328 | *88.4%* | **0.023** |  |
|  | Previously treated^‡^ | 51 | *17.8%* | 43 | *11.6%* |  |  |
| Type of TB Diagnosis | Bacteriologically confirmed | 162 | *58.9%* | 193 | *54.4%* | 0.254 |  |
|  | Clinical Diagnosis | 113 | *41.1%* | 162 | *45.6%* |  |  |
| Site of TB | Extrapulmonary | 36 | *12.5%* | 46 | *12.4%* | 0.979 |  |
|  | Pulmonary | 252 | *87.5%* | 324 | *87.6%* |  |  |
| TB Drug Sensitivity | Drug-resistant | 7 | *2.4%* | 14 | *3.8%* | 0.317^†^ |  |
|  | Drug-sensitive | 286 | *97.6%* | 359 | *96.2%* |  |  |
| TB Treatment Outcomes | Poor outcome* | 62 | *22.1%* | 56 | *15.0%* | **0.020** |  |
|  | Cure/treatment completion | 219 | *77.9%* | 317 | *85.0%* |  |  |
| *^Hypertension was defined as systolic ≥ 130 mmHg or diastolic ≥ 80 mmHg.*  *§A person with tuberculosis who has never received treatment or has only previously ever taken anti-tuberculosis drugs for less than 1 month.*  *‡Previously treated includes patients with relapse, treatment failure, loss to follow up, or transfer from another facility.*  **Poor outcome included those who died, lost to follow-up, treatment failure, and not evaluated.*  *Observations not available for the following: Smoking history: N=561, BMI: N=44, Hypertension: N=316, CD4: N=297, Patient Type: N=10, Type of TB Diagnosis: N=37; Site of TB: N=9, TB Drug Sensitivity: N=1, TB Treatment Outcomes: N=13.*  *^†^Fisher’s exact test.* | | | | | | |  |
