# Supplementary material for: TB and diabetes in Eswatini: Addressing suboptimal treatment outcomes through integrated services
Source: PLOS Glob Public Health. 2025 May 29;5(5):e0004607. doi: 10.1371/journal.pgph.0004607 (PMC12121823; doi:10.1371/journal.pgph.0004607)
Supplement: S3 Table — (DOCX) [file pgph.0004607.s003.docx]

**S3 Table**: Comparison of baseline characteristics of those with cure/treatment completion (n=317) vs. poor outcomes (n=56)

| **Patient Characteristics** | | **Cure/Treatment Completion** *(317, 85.0%)* | | **Poor Outcome** *(56, 15.0%)* | | **P-value** |  |
| --- | --- | --- | --- | --- | --- | --- | --- |
| Clinic Location | Rural | 85 | *26.8%* | 14 | *25.0%* | 0.777 |  |
|  | Urban | 232 | *73.2%* | 42 | *75.0%* |  |  |
| Sex | Female | 149 | *47.0%* | 23 | *41.1%* | 0.412 |  |
|  | Male | 168 | *53.0%* | 33 | *58.9%* |  |  |
| Age (median, IQR) | | 38 | *31-46* | 44 | *33.5-57.5* | **0.001** |  |
| Smoking History | No smoking history | 54 | *78.3%* | 3 | *75.0%* | 0.540^†^ |  |
|  | Smoking history | 11 | *15.9%* | 1 | *25.0%* |  |  |
| BMI | Normal weight (≤24.9 kg) | 243 | *78.6%* | 35 | *72.9%* | 0.374 |  |
|  | Overweight/obese (>25.0 kg) | 66 | *21.4%* | 13 | *27.1%* |  |  |
| Hypertension^ | Normal blood pressure | 135 | *81.3%* | 28 | *73.7%* | 0.289 |  |
|  | Hypertension | 31 | *18.7%* | 10 | *26.3%* |  |  |
| HIV Status | Negative | 80 | *25.2%* | 8 | *14.3%* | 0.075 |  |
|  | Positive | 237 | *74.8%* | 48 | *85.7%* |  |  |
| HIV Diagnosis | New HIV | 93 | *42.5%* | 19 | *61.3%* | **0.049** |  |
|  | Established HIV | 126 | *57.5%* | 12 | *38.7%* |  |  |
| ART Regimen | DTG-based regimen | 176 | *98.3%* | 13 | *6.7%* | 0.262^†^ |  |
|  | Non-DTG-based regimen | 3 | *1.7%* | 1 | *0.5%* |  |  |
| CD4 (median, IQR) | | 161 | *60-374* | 92 | *32-155* | 0.092 |  |
| TB Patient Type | New patient^§^ | 284 | *90.2%* | 44 | *80.0%* | **0.030** |  |
|  | Previously treated^‡^ | 31 | *9.8%* | 11 | *20.0%* |  |  |
| Type of TB Diagnosis | Bacteriologically confirmed | 173 | *57.3%* | 20 | *38.5%* | **0.012** |  |
|  | Clinical Diagnosis | 129 | *42.7%* | 32 | *61.5%* |  |  |
| Site of TB | Extrapulmonary | 34 | *10.8%* | 11 | *20.4%* | **0.047** |  |
|  | Pulmonary | 281 | *89.2%* | 43 | *79.6%* |  |  |
| TB Drug Sensitivity | Drug-resistant | 12 | *3.4%* | 1 | *1.8%* | 0.700^†^ |  |
|  | Drug-sensitive | 304 | *86.9%* | 55 | *98.2%* |  |  |
| Diabetes | New Diabetes/Pre-existing DM | 33 | *10.4%* | 17 | *30.4%* | **0.000** |  |
|  | No DM/Pre-DM | 284 | *89.6%* | 39 | *69.6%* |  |  |
| *§A person with tuberculosis who has never received treatment or has only previously ever taken anti-tuberculosis drugs for less than 1 month.*  *‡Other patients were those who were previously treated due to relapsed, treatment failure, loss to follow up, or transfer from another facility.*  **Poor outcome included those who died, were lost to follow up, treatment failure, and not evaluated.*  *Observations not available for the following: Smoking history: N=304, BMI: N=16, Hypertension: N=169, HIV Diagnosis: N=35; CD4: N=166, Patient Type: N=3, Type of TB Diagnosis: N=19; Site of TB: N=4, TB Drug Sensitivity: N=1.*  *^†^Fisher’s exact test* | | | | | | |  |
