# Supplementary material for: TB and diabetes in Eswatini: Addressing suboptimal treatment outcomes through integrated services
Source: PLOS Glob Public Health. 2025 May 29;5(5):e0004607. doi: 10.1371/journal.pgph.0004607 (PMC12121823; doi:10.1371/journal.pgph.0004607)
Supplement: S1 Text — (PDF) [file pgph.0004607.s004.pdf]

DETECT STUDY
HEALTH CARE PROVIDERS INTERVIEW GUIDE

#	Primary Question	Secondary Question
**1. Introduction and General Information**		
I would like to start our interview by asking you about your background and the work you do in this clinic.		
1.1	Please tell me how old you are (as of last birthday).	
1.2	What is the highest level of education you completed?	
1.3	Please tell me about your work at this clinic.	• What are your responsibilities?<br>• How long have you worked here?<br>• Has your position changed since you started working here? How?
1.4	Have you cared for TB patients with diabetes/prediabetes?	• Have you diagnosed and/or managed diabetes/prediabetes in TB patients?<br>• How often?
1.5	What specific training have you received on diagnosis and treatment of TB/diabetes?	**PROBE for**<br>• Preclinical training only<br>• In service training/mentorship/supervision
1.6	What kind of information and education materials, if any, have you used for TB/DM?	**PROBE for**<br>• Job aids and patient literacy materials<br>• Were they useful?
**2. Integrating prediabetes and diabetes in TB services**		
Now I would like to talk to you about the diagnosis and treatment of prediabetes and diabetes in your TB patients. I want to get your feedback about some possible ways that the TB program can develop to support you in integrating diabetes into TB services.		
2.1	Do you feel ready to deliver a prediabetes or diabetes diagnosis to TB patients?	• Can you tell me more?<br>• What tools or other support would make it easier? **PROBE** for clinical algorithm, flipchart, animated video on a tablet<br>• What type of training do you feel would be helpful?
2.2	Do you feel ready to treat diabetes in TB patients?	• Can you tell me more?<br>• What tools or other support would make it easier? **PROBE** for clinical algorithm, flipchart, animated video on a tablet<br>• What type of training do you feel would be helpful?
2.3	What types of additional information regarding diabetes/prediabetes would you like to receive?	• What type of information would be useful to you?<br>• Who would you like to deliver more information?<br>• How would you like to receive this information?
2.4	How helpful would it be to have regular visits by a nurse mentor to support your work with people with TB who have prediabetes or diabetes?	• How often would you want the visits?<br>• Can you tell me more?
2.5	We developed a brochure that can be given to patients. Can you tell me what you think about the brochure? [*SHOW THE BROCHURE*]	• Do you think that the brochure will be easily understood?<br>• What do you think about the messages in the brochure?<br>• What do you think about the images, are they clear or should be changed?<br>• How do you think patients will relate to the brochure?

		• Do you think the brochure will motivate patients to engage in a healthy lifestyle?<br>• How helpful do you think the brochure will be to providers in talking to patients?<br>• How helpful do you think the brochure will be for patients to share with their families?
2.6	Is there anything else you'd like to add?	

Thank you so much for taking the time to participate in this discussion. We really appreciate it. Your contributions will help us improve TB and diabetes services in Eswatini.

DETECT STUDY
KEY INFORMANT INTERVIEW GUIDE

#	Primary Question	Secondary Question
**1. Introduction and General Information**		
I would like to start our interview by asking you about your background and the work you do in this clinic.		
1.1	Please tell me about your work in this organization.	• What are your responsibilities?<br>• How long have you worked here?<br>• Has your position changed since you started working here? How?
1.2	What do you think about the prevalence of TB in Eswatini?	
**2. Attitudes toward diabetes**		
Now I would like to talk with you about diabetes and its potential impact on TB services.		
2.1	What are your thoughts about the prevalence of diabetes and prediabetes in Eswatini?	**PROBE for** whether<br>• Seeing more people at risk for diabetes.<br>• Seeing more people with diabetes.<br>• Changes in prevalence of diabetes and prediabetes in recent years.
2.2	What are your thoughts about routine and ongoing TB screening for people with diabetes/prediabetes?	• What are the benefits of TB screening for people with diabetes?<br>• What are the challenges to TB screening for people with diabetes?<br>• Do you think TB screening is practiced routinely for people with diabetes/prediabetes?<br>• Tell me about a time when TB screening was not routinely implemented for people with diabetes/prediabetes.<br>• What do you think we should do to make sure that TB screening is routinely done?<br>• Can you tell me more?
2.3	What are your thoughts about diabetes screening for TB patients?	• What are the benefits of diabetes screening for TB patients?<br>• What are the challenges to diabetes screening for TB patients?<br>• Can you tell me more about this?
**3. Managing diabetes and prediabetes in TB Patients**		
Now I would like to talk to you about the management of diabetes and prediabetes in TB patients.		
3.1	What are your thoughts about the effectiveness of managing diabetes or prediabetes with lifestyle changes such as changes to diet and exercise?	• Can you tell me more?
3.2	What are your thoughts about the effectiveness of diabetes medications and glycemic control?	• Can you tell me more?<br>**PROBE for** whether<br>• Think the diabetes treatment is complex.
3.3	What do you recommend to improve diabetes treatment strategies for your TB patients?	**PROBE for**<br>• Diagnosis, treatment and follow up<br>• Continuous medical education
**4. Potential intervention components**		
Finally, I want to get your feedback about some possible ways that the TB program can develop to support TB patients who have diabetes or prediabetes.		

4.1	What type of support do you think would be helpful to provide TB patients regarding diabetes/prediabetes?	**PROBE for**<br>• Educational program on diabetes and its management:<br>o one-to-one or in a group<br>o face-to-face or virtual<br>• Phone messaging, such as SMS or Whatsapp<br>• Support program:<br>o one-to-one or in a group<br>o face-to-face or virtual<br>• Other
4.2	What would it take to get TB patients to participate in a program that helps prevent them from developing or controlling their diabetes?	• Can you tell me more?
4.3	What type of activities would you like a diabetes program to include?	**PROBE for**<br>• Weighing patients in each meeting<br>• Exercise class that includes activities such as jumping rope or jumping jacks<br>• Cooking class that will teach patients healthy cooking<br>• Teaching patients how to shop for healthy foods<br>• Other
4.4	What do you think is the ideal duration of a diabetes program?	• How frequently would you want to have program meetings?<br>• How many meetings would you like to have in the program?<br>• How long would an ideal program meeting be?
4.5	Where would you like TB patients to receive this program -- in TB clinic, primary care clinic, or somewhere else?	• Can you tell me more?
4.6	What is the best place to have program meetings -- in the community or in the clinic?	• Can you tell me more?
4.7	Is there anything else you'd like to add?	

Thank you so much for taking the time to participate in this discussion. We really appreciate it. Your contributions will help us improve TB and diabetes services in Eswatini.

Self-Administered Assessment:

What is your sex? ____

What is your current age? ____

What is the highest level of education you completed? ______________________________

How long have you been working in the TB/AIDS/NCD program? ______________________________

Have you had direct contact with TB or diabetes patients in a clinical, educational, and/or counseling role? ____________
____

Did you ever have TB disease yourself? ____
