# Supplementary material for: TB and diabetes in Eswatini: Addressing suboptimal treatment outcomes through integrated services
Source: PLOS Glob Public Health. 2025 May 29;5(5):e0004607. doi: 10.1371/journal.pgph.0004607 (PMC12121823; doi:10.1371/journal.pgph.0004607)
Supplement: S2 Text — (PDF) [file pgph.0004607.s005.pdf]

Variable	Name	Response
A01	Sex	1 = Male<br>2 = Female
A02	Age at enrollment	Age in years
A03	Clinic location	1 = Urban<br>2 = Rural
B01	BMI category at TB treatment initiation	1 = Underweight/Healthy weight<br>2 = Overweight/Obese
B02	Hypertension at TB treatment imitation	1 = Normal blood pressure<br>2 = Hypertension
B03	Date of HbA1c collection	Date (MM/DD/YY)
B04	HbA1c at TB treatment initiation	HbA1c in %
B05	HIV status	1 = HIV positive<br>2 = HIV negative
B06	HIV diagnosis	1 = New HIV<br>2 = Established HIV
B07	ART regimen	1 = DTG-based regimen<br>2 = Non-DTG-based regimen
B08	Most recent CD4 count	CD4 (per µL)
B09	Smoking history	1 = No smoking history<br>2 = Smoking history
C01	Type of TB diagnosis	1 = Bacteriologically confirmed<br>2 = Clinical diagnosis
C02	Site of TB diagnosis	1 = Pulmonary<br>2 = Extrapulmonary
C03	TB patient type	1 = New patient<br>2 = Previously treated
C04	TB drug sensitivity	1 = Drug-resistant<br>2 = Drug-sensitive
C05	TB treatment outcome	1 = Poor outcome<br>2 = Cure/treatment completion
C06	Diabetes status	1 = no DM/pre-DM<br>2 = DM
C07	Pre-existing DM	1 = yes
